# Zinc-Based Nanoparticles Reduce Bacterial Biofilm Formation

**DOI:** 10.1128/spectrum.04831-22

**Published:** 2023-02-28

**Authors:** Rafael Bianchini Fulindi, Juliana Domingues Rodrigues, Thulio Wliandon Lemos Barbosa, Ariana D. Goncalves Garcia, Felipe de Almeida La Porta, Sebastião Pratavieira, Leila Aparecida Chiavacci, João Pessoa Araújo Junior, Paulo Inácio da Costa, Luis R. Martinez

**Affiliations:** a Departments of Clinical Analysis, São Paulo State University (UNESP), Araraquara, São Paulo, Brazil; b Departments of Drugs and Medicines, School of Pharmaceutical Sciences, São Paulo State University (UNESP), Araraquara, São Paulo, Brazil; c Department of Oral Biology, University of Florida College of Dentistry, Gainesville, Florida, USA; d Department of Chemistry, Federal Technological University of Paraná (UTFPR), Londrina, Paraná, Brazil; e São Carlos Physics Department, University of São Paulo, São Carlos, São Paulo, Brazil; f Biotechnology Institute, São Paulo State University, Botucatu, São Paulo, Brazil; g Emerging Pathogens Institute, University of Florida, Gainesville, Florida, USA; h Center for Immunology and Transplantation, University of Florida, Gainesville, Florida, USA; i Center for Translational Research in Neurodegenerative Disease, University of Florida, Gainesville, Florida, USA; USDA - San Joaquin Valley Agricultural Sciences Center

**Keywords:** antimicrobial activity, bacteria, biofilms, nanoparticles, zinc oxide, zinc sulfide

## Abstract

Biofilm formation is important for microbial survival in hostile environments and a phenotype that provides microorganisms with antimicrobial resistance. Zinc oxide (ZnO) and Zinc sulfide (ZnS) nanoparticles (NPs) present potential antimicrobial properties for biomedical and food industry applications. Here, we aimed to analyze, for the first time, the bactericidal and antibiofilm activity of ZnS NPs against Staphylococcus aureus, Klebsiella oxytoca, and Pseudomonas aeruginosa, all medically important bacteria in developed countries. We compared ZnS NPs antimicrobial activity to ZnO NPs, which have been extensively studied. Using the colorimetric XTT reduction assay to observe the metabolic activity of bacterial cells and the crystal violet assay to measure biofilm mass, we demonstrated that ZnS and ZnO had similar efficacy in killing planktonic bacterial cells and reducing biofilm formation, with S. aureus being more susceptible to both therapeutics than K. oxytoca and P. aeruginosa. Crystal violet staining and confocal microscopy validated that Zn NPs inhibit biofilm formation and cause architectural damage. Our findings provide proof of principle that ZnS NPs have antibiofilm activity, and can be potentially used in medical and food industry applications, such as treatment of wound infections or package coating for food preservation.

**IMPORTANCE** Zinc (Zn)-based nanoparticles (NPs) can be potentially used in medical and food preservation applications. As proof of principle, we investigated the bactericidal and antibiofilm activity of zinc oxide (ZnO) and zinc sulfide (ZnS) NPs against medically important bacteria. Zn-based NPs were similarly effective in killing planktonic and biofilm-associated Staphylococcus aureus, Klebsiella oxytoca, and Pseudomonas aeruginosa cells. However, S. aureus was more susceptible to these investigational therapeutics. Although further studies are warranted, our findings suggest the possibility of future use of Zn-based NPs in the treatment of skin infections or preservation of food.

## INTRODUCTION

Biofilm formation by medically-important bacteria represents a problem for the population health, because this phenotype is a critical factor for microbial survival, colonization, and antibiotic resistance (1). Annually, bacterial infections affect ~300 million people globally, including killing ~2 million children (2), and having an associated cost of ~$24 billion dollars in developed countries (3). Patients in intensive care are particularly vulnerable to multidrug-resistant bacterial infections, especially those using nasogastric tubes (4). About 50% of nosocomial infections are related to patients with indwelling devices used for medical treatment purposes, such as catheters, cardiac pacemakers, joint replacements, dental prostheses, prosthetic heart valves, and contact lenses (5). These devices provide an ideal surface for bacterial cell attachment. Biofilm-forming bacteria cause a large number of infections, including endocarditis, osteomyelitis, sinusitis, urinary tract infections, chronic prostatitis, periodontitis, chronic lung infection in patients with cystic fibrosis, and middle ear infections (5). Moreover, biofilms also cause an enormous economic loss to the food industry every year (6). For example, food spoilage and degradation also compromise food safety, which is a major priority in today's globalized market, with worldwide transportation and consumption of fresh and minimally processed food.

Staphylococcus aureus, Klebsiella oxytoca, and Pseudomonas aeruginosa, are listed as high priority bacteria by World Health Organization due to their antibiotic resistance (3), and their biofilm formation ability, representing a threat in medicine and food industry. S. aureus is a Gram-positive human commensal that persistently colonizes 80% of individuals with nosocomial infections (7). S. aureus forms biofilms and produces multiple virulence factors, such as extracellular enzymes (e.g., coagulase A) and toxins (e.g., Panton-Valentine Leukocidin) (8). Furthermore, S. aureus adheres to host tissue (e.g., bone and heart valves) and medical implants (e.g., catheters, prosthetic joints, and pacemakers) forming biofilms, which play an important role in disease establishment and persistence (9). For example, infections caused by antibiotic-resistant S. aureus alone cost the United States health care systems $1.7 billion dollars per year, and kills ~11,000 patients (10).

Gram-negative bacteria, such as P. aeruginosa and K. oxytoca, have become impervious to commonly used antibiotics causing infections very difficult to treat resulting in high mortality. P. aeruginosa has a broad virulence factor armamentarium, including a robust biofilm formation ability that causes serious health problems in immunocompromised patients, including those with cystic fibrosis or are wounded/burned (11). K. oxytoca has frequent commensal occurrence in the nasopharyngeal and intestinal tract of humans causing opportunistic disease in immunocompromised patients (12). Outbreaks of multidrug-resistant K. oxytoca have occurred in hospitals around the world (13), particularly in long-term-care facilities and intensive care units (14), and handwashing stations have been identified as a possible environmental reservoir (15). Therefore, novel therapeutic and treatment strategies are urgently needed to combat infections by Gram-negative bacteria, especially those with natural abilities to form biofilms.

Among the possibilities to combat bacterial biofilm formation, inorganic metal-based nanoparticles (NPs) such as silver, titanium, gold, copper, iron, and zinc (Zn) have been explored (16). They exhibit antiseptic and antimicrobial properties with potential for biomedical applications. For example, zinc oxide (ZnO) NPs have anti-microbial and -biofilm activity on a wide range of Gram-positive and -negative bacteria (17), which can be ideal to be used in the food packing industry and agriculture (18). In contrast, the application of Zn sulfide (ZnS) as an antimicrobial or antibiofilm agent has not been reported. Zn NPs inhibit bacterial viability and proliferation via generation of reactive oxygen species (ROS) causing damage to cellular components, i.e., lipids, proteins, and DNA or release of Zn^2+^ ions inducing toxicity through apoptosis (19). Here, we describe the efficacy of ZnS NPs on bacterial planktonic and biofilm inhibition and compared it to ZnO. We report that ZnS NPs kill single bacterial cells and reduce, considerably, biofilm formation, providing a proof of principle and suggesting the possibility of being promisingly applied in medicine and the food industry.

## RESULTS

### Planktonic bacteria are susceptible to Zn NPs.

**FIG 1:**
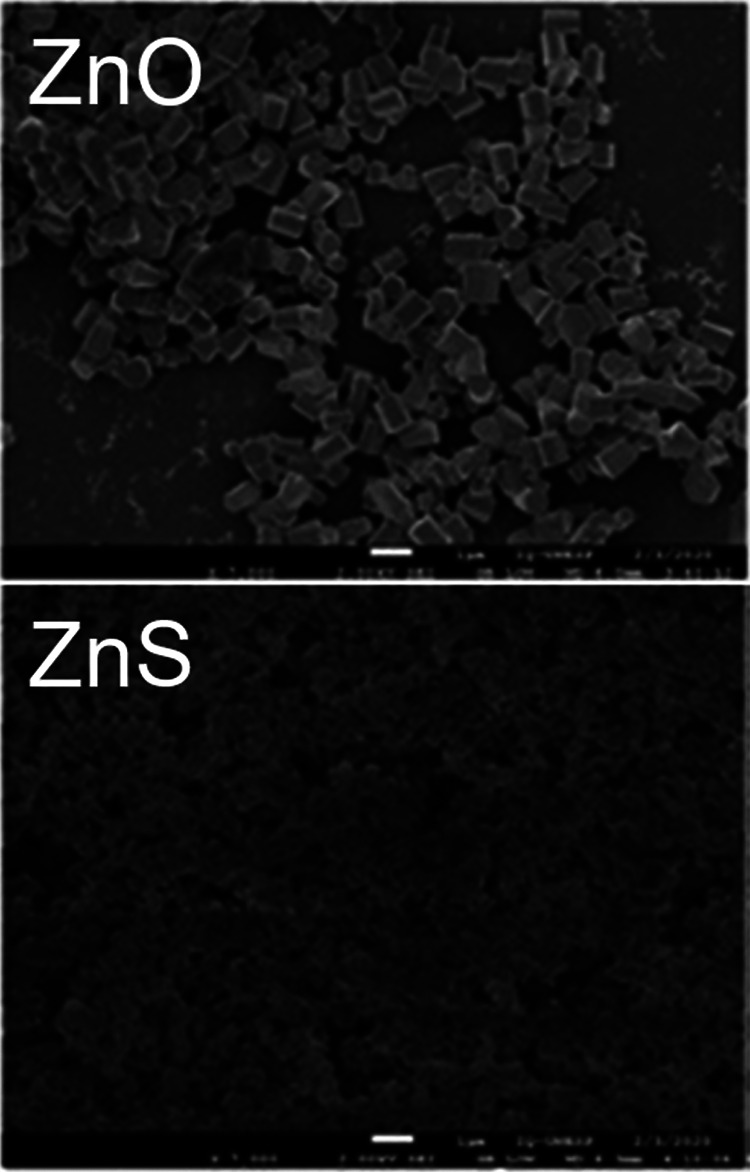
Analysis of ZnO and ZnS nanoparticles (NPs) by scanning electron microscopy (SEM). SEM photographs show zinc oxide (ZnO, upper panel) and zinc sulfide (ZnS, lower panel) nanoparticles (NPs). ZnO NPs consists of uniformed rectangles of ~500 nm. ZnS NPs exhibited smaller structures of ~150 nm. Scale bar: 1 μm.

We developed and generated ZnO and ZnS NPs, as described by La Porta et al. (20) and Savu et al. (21), respectively. First, we used scanning electron microscopy (SEM) to characterize the shape and size of these NPs (Fig. 1). ZnO presents a hexagonal prism or wurtzite structure of ~500 nm in size (Fig. 1, upper panel), while ZnS exhibits a combination of smaller cubic and wurtzite shape structures of ~150 nm (Fig. 1, lower panel). Our Zn NPs exhibited the expected shapes and sizes, thus, validating the synthesis protocols previously described (20, 21). Then, we evaluated the antimicrobial activity of ZnO and ZnS NPs against planktonic S. aureus, K. oxytoca, and P. aeruginosa (Table 1). We determined the MIC and minimal bactericidal concentration (MBC) for each bacterial strain using the XTT reduction assay and CFU counts, respectively. K. oxytoca and P. aeruginosa were more resistant than S. aureus to the antimicrobial activity of Zn NPs. MIC/MBC for S. aureus were 1 mg/mL for both Zn NPs and MIC/MBC for Gram-negative bacteria were 4 mg/mL. These results indicate that single microbial cells are sensitive to ZnO and ZnS NP exposure.

**TABLE 1:** Minimum inhibitory (MIC) and bactericidal (MBC) concentrations of ZnO and ZnS NPs against bacteria (*n* = 3)

Bacteria	MIC (mg/mL)		MBC (mg/mL)	
	ZnO	ZnS	ZnO	ZnS
S. aureus	1.0	1.0	1.0	1.0
K. oxytoca	4.0	4.0	4.0	4.0
P. aeruginosa	4.0	4.0	4.0	4.0

### Zn NPs reduce biofilm biomass.

**FIG 2:**
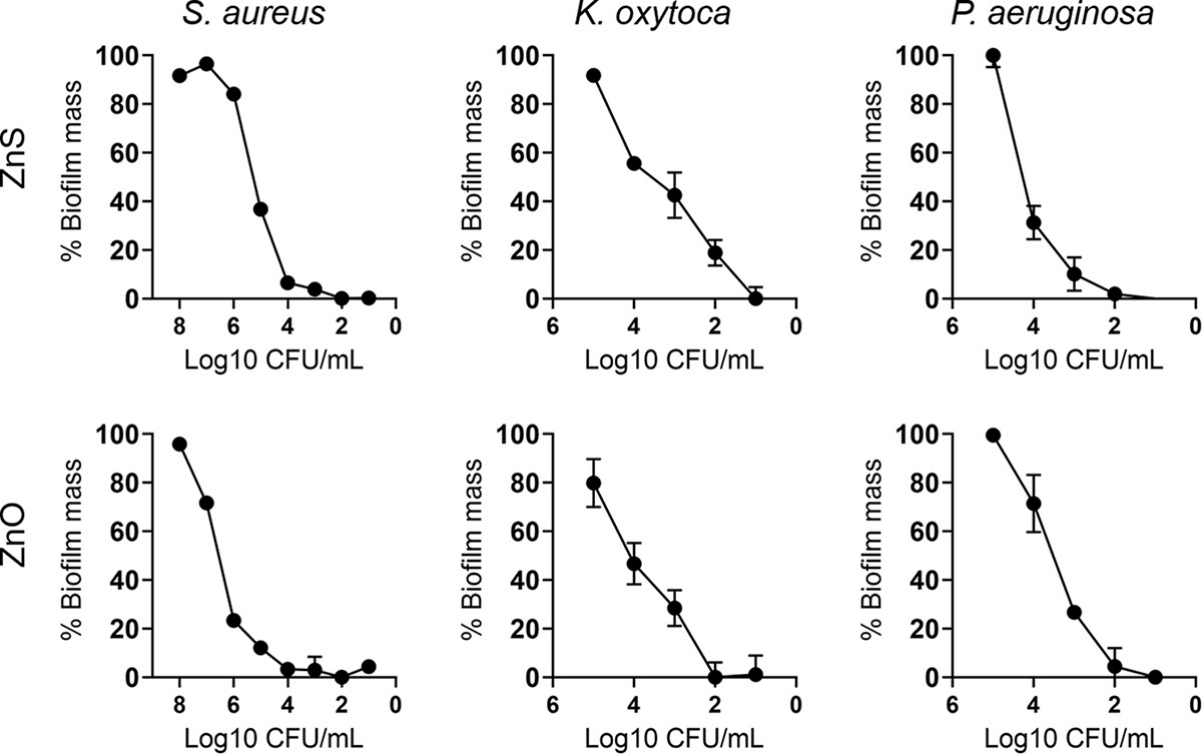
Zn NPs decrease Staphylococcus aureus, Klebsiella oxytoca, and Pseudomonas aeruginosa biofilm biomass as determined by the crystal violet assay. Crystal violet solubilization assay with 30% acetic acid in distilled water, with inoculum of S. aureus, K. oxytoca, and P. aeruginosa added together with ZnO and ZnS, and grown in 96-well polystyrene microtiter plates for 24 h. Microbial biofilms were grown alone or with 0.5 mg/mL (S. aureus) or 2 mg/mL (K. oxytoca or P. aeruginosa). All experiments were performed three times (representative data shown), and similar results were obtained each time.

**FIG 3:**
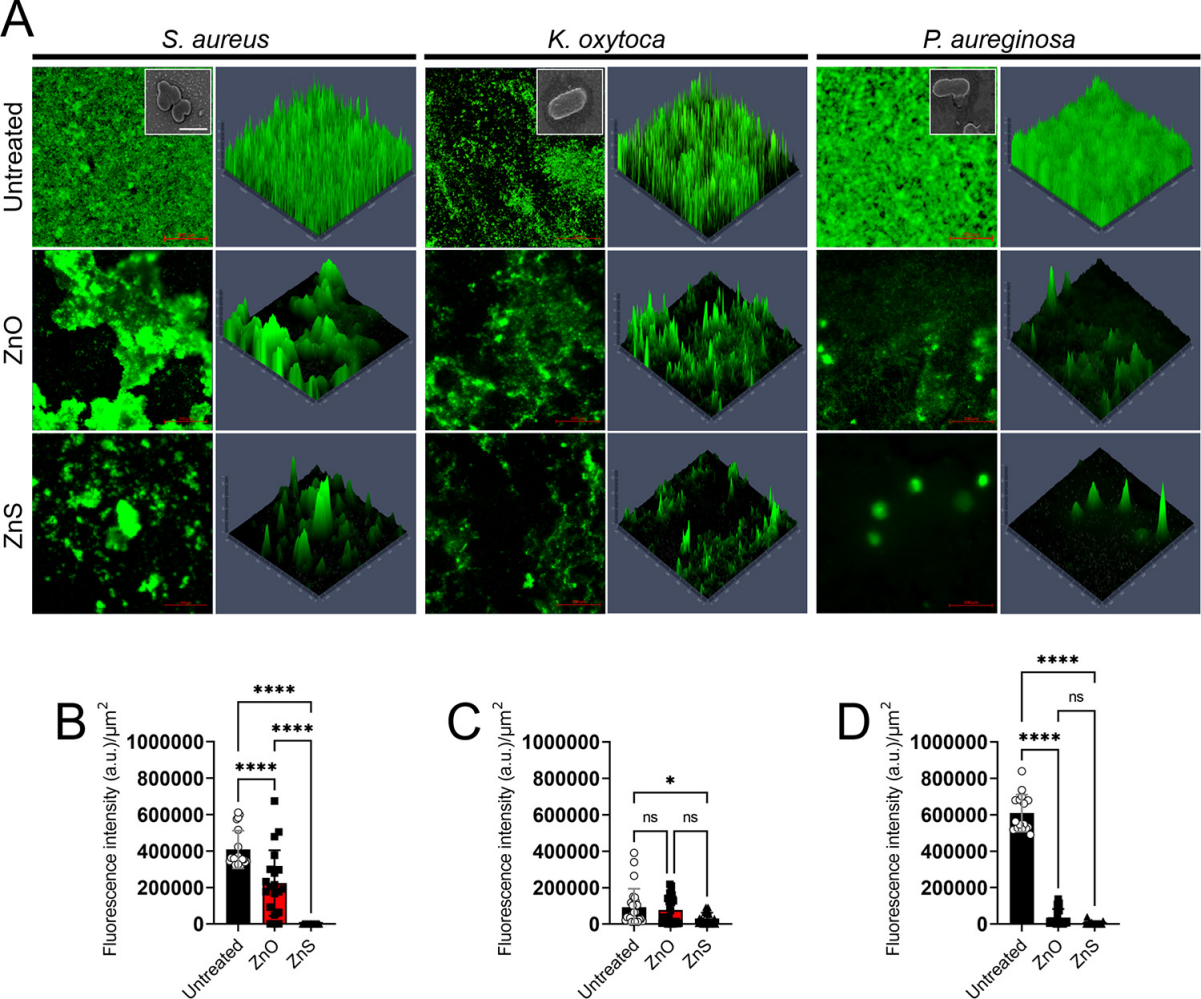
Confocal microscopy of bacterial biofilms after 24 h of growth in the presence ZnO or ZnS NPs. (A) Bacterial biofilms were grown alone (untreated) or with 0.5 mg/mL (S. aureus) or 2 mg/mL (K. oxytoca or P. aeruginosa). Representative images of untreated-, ZnO-treated, or ZnS-treated biofilms (green [SYTO9]) are shown. The thickness and morphology of the bacterial biofilms can be observed in the *Z*-stack reconstruction. The photographs were taken at a magnification of ×63. Scale bar: 200 μm. Insets show SEM of single bacterial cells taken at a magnification of ×100. Scale bar: 1 μm. The fluorescent intensity/μm^2^ of (B) S. aureus, (C) K. oxytoca, and (D) P. aeruginosa biofilm images of bacteria grown in absence or presence of Zn NPs was quantified using ImageJ software within a defined 100 μm x 100 μm region of interest (ROI). For (B to D), bars and error bars denote the means and standard deviations (SDs), respectively. Each symbol represents an individual ROI (*n *= 20 for S. aureus; *n *= 25 for K. oxytoca; *n *= 16 for P. aeruginosa). Asterisks denote *P* value significance (*, *P < *0.05; and ****, *P < *0.0001) calculated using analysis of variance (ANOVA), and adjusted by use of the Tukey’s *post hoc* analysis. ns denotes not statistically significant comparisons. a.u. signifies arbitrary units. These experiments were performed twice, and similar results were obtained each time. All the results combined are presented.

Biofilm formation on 96-well microtiter plates was compared among the different bacterial strains using the crystal violet method, which stains both cellular and matrix component of biofilms (Fig. 2). Like planktonic bacterial cells, Zn NPs significantly decreased mature biofilm (24 h) biomass for all the strains, especially for S. aureus. ZnO-treatment of S. aureus biofilms had 23% biomass when the initial bacterial density was 10^6^ CFU/mL (Fig. 2). Biofilms grown with ZnS had substantially reduced biomass (38%) when the initial microbial density was 10^5^ CFU/mL. Zn NPs completely reduced S. aureus biofilm biomass at bacterial cell concentrations ≤10^4^ CFU/mL. ZnS and ZnO reduced K. oxytoca biofilm biomass to ~50% at ≤10^4^ CFU/mL. The biomass of K. oxytoca biofilms were completely reduced at cell densities ≤10^2^ and ≤10^1^ CFU/mL after treatment with ZnO and ZnS, respectively. Likewise, P. aeruginosa showed higher susceptibility to ZnS NPs than K. oxytoca, evincing 30% biofilm biomass at density ≤10^4^ CFU/mL and less than 10% at ≤10^3^ CFU/mL. P. aeruginosa biofilm biomass was almost completely reduced at a density ≤10^2^ CFU/mL. To confirm the ability of Zn NPs to inhibit bacterial biofilm formation, confocal microscopy was used to monitor S. aureus, K. oxytoca, and P. aeruginosa biofilm formation for 24 h in absence (untreated) or presence of ZnO or ZnS (Fig. 3). Since S. aureus is more sensitive (e.g., MIC and MBC) to Zn NPs, we treated the cocci with a lower concentration than for K. oxytoca and P. aeruginosa (0.5 mg/mL versus 2 mg/mL). All untreated bacteria grown formed robust biofilms characterized by uniformed cell-matrix distribution across the microscopic field. SEM shows individual bacterial cells, clustered cocci for S. aureus, and rod-shape for K. oxytoca and P. aeruginosa (Fig. 3A, insets). Untreated S. aureus, K. oxytoca, and P. aeruginosa formed biofilms of a depth of 120, 80, and 160 μm, respectively (Fig. 3A). ZnO and ZnS NPs substantially reduced the depth of the bacterial biofilms and their distribution across the fields. ZnS NPs-treated bacteria exhibited more biofilm reduction relative to ZnO NPs. Interestingly, although untreated P. aeruginosa showed a denser and deeper biofilm than the other bacteria, treatment with either Zn NPs considerably reduced this Gram-negative bacterium biomass. To validate the image visualization, we analyzed and quantified the fluorescent intensity/μm^2^ of the biofilm images of bacteria grown in absence or presence of Zn NPs (Fig. 3B to D). Untreated S. aureus biofilms demonstrated significantly higher fluorescent intensity/μm^2^ than biofilms grown with ZnO (*P < *0.0001) and ZnS (*P < *0.0001) (Fig. 3B). In contrast, ZnS-treated S. aureus biofilms exhibited the lowest fluorescent intensity/μm^2^. Untreated K. oxytoca biofilms displayed significantly higher fluorescent intensity/μm^2^ than ZnS-treated biofilms (*P < *0.05), although no difference was observed between ZnO-treated biofilms and the untreated or ZnS-treated conditions (Fig. 3C). The fluorescent intensity/μm^2^ of P. aeruginosa biofilms was similarly reduced after treatment with either ZnO or ZnS NPs (*P < *0.0001) (Fig. 3D). Our findings indicate that Zn NPs are effective in inhibiting biofilm formation, suggesting a possible application in medicine and food preservation.

## DISCUSSION

Bacterial cutaneous infections, antibiotic resistance, food poisoning, and food spoilage are prevalent in developed countries exacerbating morbidity and mortality. There is an urgent need for new therapeutics to reduce these incidences and enhance people’s quality of life. Here, we reported, for the first time, the efficacy of ZnS NPs against bacteria of medical importance due to their ability to form biofilms and become resistant to commonly used antibiotics. We also validated the effectiveness of ZnO NPs, which have been extensively characterized (22, 23). We obtained similar size and shape ZnS NPs as described in (20), which consisted of blended nano cubic and wurtzite structures. MIC and MBC results indicate that planktonic cells of the Gram-positive S. aureus strain are more susceptible to Zn NPs than Gram-negative K. oxytoca and P. aeruginosa strains. It is possible that differences in the cell surface between Gram-positive and Gram-negative bacteria influence the antimicrobial activity of the Zn NPs. For example, the presence of, or alterations to, the double membrane (24), having a periplasmic space, production and secretion of proteins (23, 25), and overexpression of efflux pumps (26, 27), or porins (28) in Gram-negative bacteria may reduce the Zn NPs antimicrobial or antibiofilm activity. P. aeruginosa produces pyocyanin to protect itself against NPs by inactivating ions produced or released (23). Moreover, P. aeruginosa strain CCM 3955 overexpresses a flagellin matrix that causes the agglomeration of silver NPs to avoid direct contact, which is bactericidal (25). K. oxytoca shows high resistance to diverse metals, including Zn, and robust biofilm formation in wastewater (29), providing a plausible explanation to the persistence of this bacterium in handwashing reservoirs and its ability to cause disease in immunocompromised individuals in contact with these environmental sources. It is also important to explain that the MIC and MBC concentrations of Zn NPs that inhibited and killed planktonic bacteria were the same, because the XTT reduction assay and CFU determinations typically correlate, even though either approach measure different parameters, such as metabolic activity and viability.

Two main mechanisms of Zn NPs-mediated bacterial growth inhibition have been proposed, including ROS production and release of Zn^2+^ ions. For example, ZnO damages bacteria and inhibits biofilm formation by increasing ROS, such as hydroxyl superoxide anion (O_2_^•−^), hydroxyl free radicals (·OH), and hydrogen peroxide (H_2_O_2_) that can lead to the destruction of cellular components, such as DNA, proteins, and lipids through oxidative stress (30). Another mechanism involves the production of Zn^2+^ ions upon dissolution of ZnO NPs (22). Zn^2+^ ions prolong the lag phase of growth and interfere in biochemical processes, such as glycolysis, active and passive proton transport across the membrane, acid tolerance, and quorum sensing (31). These mechanisms are important to further elucidate how ZnS NPs kill bacteria.

The crystal violet staining and confocal microscopy demonstrated that bacterial biofilm formation was significantly reduced when bacteria was cultured with Zn NPs, corroborating the results obtained in MIC and MBC assays. In addition to the bactericidal activity of Zn NPs, it is conceivable that Zn NPs interfere with cell-to-cell interactions by altering their surface negative charge or zeta potential, inhibiting adhesion to the substrate surface. Similarly, Zn NPs may alter gene expression, especially genes associated with adhesion structures, such as fimbriae, pilus, or capsule (32), quorum sensing (33), or extracellular matrix synthesis (32). The adhesion and capsular encoding genes are critically associated with biofilm formation among K. oxytoca isolates (32). ZnO NPs stop capsular gene expression in Klebsiella pneumoniae clinical isolates (34). ZnO NPs inhibit quorum sensing (35) and its dependent virulence factors and biofilm formation in P. aeruginosa isolates (33). In addition, ZnO NPs inhibit S. aureus quorum sensing (e.g., accessory gene regulator, *agr*)- and biofilm formation (e.g., intercellular adhesion A, *icaA*)-associated genes (36). Since we found that ZnS NPs have similar antibiofilm activity than ZnO NPs, it is likely that both damage and kill bacteria using comparable mechanisms. Regardless, Zn NPs can be used in surface coating applications to prevent biofilm formation, such as catheters, used in the medical field or antimicrobial agents, or applied to the food industry, reducing non-deposition areas. Since bacterial resistance has not been described in Zn NPs (at least not yet), the fact that they are easy to produce, inexpensive, and biocompatible molecules make them very attractive for future development.

## MATERIALS AND METHODS

### ZnO and ZnS NPs.

The ZnS and ZnO NPs were synthesized by the Institute of Chemistry of the São Paulo State University - UNESP. ZnS NPs were synthesized by the microwave-assisted solvothermal method, as previously described (20). Briefly, 7.34 mmol of zinc acetate and 15.44 mmol of tetrabutylammonium hydroxide were dissolved in 25 mL of ethylene glycol (EG) and heated to 100°C (solution 1). Concurrently, 7.34 mmol of thiourea were diluted separately in another 25 mL of EG (solution 2). Under vigorous magnetic stirring, solution 1 was then quickly mixed with solution 2. The final pH of the solution was 10. Then, the mixed solution was transferred to a Teflon autoclave (Techinstro) that was sealed and placed inside a residential microwave-solvothermal system (2.45 GHz, maximum power 800 W) at 140°C for 1 min. The resultant solution was flushed with deionized water and ethanol multiple times to neutralize the pH of the solution (~7), and the precipitates were finally collected, which were followed by heat treatment at 70°C for 24 h. Similarly, ZnO NPs were synthetized using the hydrothermal method reported previously in (21). ZnO nanostructures were made by using aqueous solutions of zinc nitrate and hexamethylenetetramine as precursors. The reagents were dissolved in distilled water (dH_2_O) at room temperature (RT), with the template solution being slowly added to the zinc solution under continuous stirring. The resulting transparent solution was placed in a polytetrafluorethylene-lined stainless-steel pressure vessel. The substrates were suspended in the solution with the zinc-coated surface facing the bottom of the flask. The hydrothermal process was performed in a hot Vaseline bath at 110°C for a 6 h synthesis time under continuous stirring of solution. The pressure vessel was then removed from the hot bath and allowed to cool down naturally (in air), or immersed in a RT Vaseline bath in order to increase the heat-transfer rate. After 1 min immersed in the room-temperature Vaseline bath, the final temperature reached was 50°C. The nanostructured deposits were washed several times with dH_2_O and ethanol and dried in an oven at 75°C. The NPs were characterized by SEM analysis.

### SEM.

SEM analyses were performed to characterize Zn NPs shape and size and bacterial shape. After dehydration, the samples were placed in a vacuum desiccator until analysis. Each sample was coated with gold by sputtering for 20 sec under pressure of 2 × 10-1 mBar, and examined in the high-resolution SEM JEOL JSM-7500F (Jeol USA) with PC-SEM v 2,1,0,3 operating software, equipped with secondary electron backscattered detectors.

### Bacteria.

S. aureus (Gram-positive; ATCC 25923), K. oxytoca (Gram-negative; ATCC 13182), and P. aeruginosa (Gram-negative; ATCC 27853) strains were used and grown in Muller-Hinton agar (Kasvi). Microbial suspensions were prepared from a single colony grown overnight in fresh medium at 37°C, and adjusted to an optical density (625 nm) equivalent to 10^8^ CFU per milliliter (CFU/mL) using a spectrophotometer, and according to the protocol of the Clinical and Laboratory Standards Institute (CLSI).

### Determination of the MICs and MBCs.

The MICs were determined by the XTT reduction (2,3-Bis (2-methoxy-4-nitro-5-sulfophenyl)-5-[(phenylamino)carbonyl]-2H-tetrazolium hydroxide) assay to evaluate the effect of Zn NPs on microbial metabolism. Briefly, we used a microdilution method in 96-microtiter well plates, according to the M7-A10 protocol of the CLSI (37) that was modified by using Mueller-Hinton medium. A serial dilution of microbial cells was performed in Mueller-Hinton broth starting with a standardized 0.5 McFarland [optical density 625 nm (OD _625 nm_)] or 10^8^ CFU/mL, while maintaining a constant concentration of the NPs of 1.0 mg/mL for S. aureus, and 4.0 mg/mL for K. oxytoca, and P. aeruginosa. Afterwards, the microtiter plates were incubated at 37°C for 24 h. Then, aliquots of 50 μL of an XTT tetrazolium salt solution (1 mg/mL in phosphate-buffered saline [PBS]; Sigma) and 4 μL of a menadione solution (1 mM in acetone; Sigma) were added to each well of a microtiter plate containing Zn NPs-treated bacteria, followed by their incubation at 37°C for 5 h. The electron transport system in the cellular membrane of live bacteria reduces XTT tetrazolium salt to XTT formazan, resulting in a colorimetric change that correlates with cell viability (38). The colorimetric change was measured using a microtiter plate reader at 492 nm. The lowest concentration that presented a red-orange coloration after incubation with XTT indicated the MIC. After the determination of the optimal bacterial concentrations (S. aureus, 10^4^ CFU/mL; K. oxytoca and P. aeruginosa, 10^3^ CFU/mL) for our studies, an MBC assay was performed, where the bacterial density was kept constant, and the NPs were serially diluted in a microtiter plate (range: 0.0078-1 mg/mL for S. aureus and 0.0031 to 4 mg/mL for K. oxytoca and P. aeruginosa). The MBC represented the lowest Zn NP concentration, where colonies did not grow after subculturing the samples from the microplates on petri dishes with Mueller-Hinton agar incubated at 37°C for 24 h. These experiments were performed in triplicate.

### Biofilm formation.

Two hundred microliters of a suspension with 10^3^ (K. oxytoca or P. aeruginosa) or 10^4^ (S. aureus) cells in Muller-Hinton broth (Kasvi) alone, or with Zn NPs (0.5 mg/mL, S. aureus or 2 mg/mL, K. oxytoca or P. aeruginosa) was added to individual wells of polystyrene 96-well plates. As a control, the initial inoculum of each bacterial species was serially diluted and plated on Muller-Hinton agar to corroborate having the same number of microbial cells per condition. The plates were incubated at 37°C in a 5% CO_2_ aerobic atmosphere, and biofilms were formed over 24 h. After incubation, the medium was gently aspirated, and biofilms were gently washed three times with 200 μL of PBS to remove non-adhered bacteria. Bacteria that remained attached to the plastic surface were considered true biofilms. Biofilm formation was verified by crystal violet assay and confocal microscopy. All assays were carried out in triplicate.

### Crystal violet assay.

The crystal violet assay was performed to assess the efficacy of Zn NPs in reducing S. aureus, K. oxytoca, and P. aeruginosa biofilm biomass by modifying protocols described in (39). Twenty-four h biofilms were grown alone or with 0.5 mg/mL (S. aureus) or 2 mg/mL (K. oxytoca or P. aeruginosa), and gently washed with PBS to remove non-adherent bacteria. A solution of 0.1 % crystal violet was added for 15 min. Wells containing the biofilms were rinsed three times with dH_2_O, shaken against a paper towel to remove all excess dye, and air-dried. Then, a suspension of 30 % acetic acid in dH_2_O was added to each well containing biofilms to solubilize the crystal violet (Fisher Scientific), and the plate was incubated at RT for 15 min. Finally, solubilized crystal violet was measured in a microtiter reader at 550 nm using 30 % acetic acid in dH_2_O as negative control. These experiments were performed in triplicate, and the obtained results were reported as mean ± standard deviation.

### Confocal microscopy.

The architecture of biofilms was examined using the Live/Dead biofilm viability kit (Invitrogen) and confocal microscopy. Briefly, biofilms were grown for 24 h in 35-mm glass-bottom culture dishes (MatTek Corp.), alone or with 0.5 mg/mL (S. aureus) or 2 mg/mL (K. oxytoca or P. aeruginosa); rinsed three times with PBS; and incubated for 30 min at RT in 2 mL of dH_2_O containing the fluorescent stain SYTO9 (6 μL; excitation wavelength, 500 nm; emission wavelength, 535 nm), with protection from light. The dishes were then rinsed three times with dH_2_O to remove excess stain. Microscopic examinations of biofilms formed in glass-bottom plates were performed using an inverted Zeiss LSM 780 confocal laser scanning microscope. To determine the structure and thickness of the biofilms, a series of horizontal (x-y) optical sections with a thickness of 1.175 μm were taken throughout the full length of the biofilm using a 63× objective. Confocal images of green fluorescence were recorded simultaneously using a multichannel mode. The fluorescent intensity/μm^2^ of each biofilm image was quantified using ImageJ V5.3 software (NIH) within a defined 100 μm x 100 μm region of interest. *Z*-stack images and measurements were corrected by utilizing Zeiss Zen Lite software in the deconvolution mode.

### Statistical analyses.

All data were subjected to statistical analysis using Prism 9.5 (GraphPad Software). *P* values for multiple comparisons were calculated by analysis of variance (ANOVA) and adjusted using the Tukey’s multiple comparison test. *P* values of <0.05 were considered significant.

## References

[1] Hall-Stoodley L, Costerton JW, Stoodley P. 2004. Bacterial biofilms: from the natural environment to infectious diseases. Nat Rev Microbiol 2:95–108. doi:10.1038/nrmicro821. 15040259

[2] Minarini L, de Andrade LN, De Gregorio E, Grosso F, Naas T, Zarrilli R, Camargo I. 2020. Editorial: antimicrobial resistance as a global public health problem: how can we address it? Front Public Health 8:612844. doi:10.3389/fpubh.2020.612844. 33282821 PMC7689264

[3] Antimicrobial Resistance C. 2022. Global burden of bacterial antimicrobial resistance in 2019: a systematic analysis. Lancet 399:629–655. doi:10.1016/S0140-6736(21)02724-0. 35065702 PMC8841637

[4] Archer NK, Mazaitis MJ, Costerton JW, Leid JG, Powers ME, Shirtliff ME. 2011. *Staphylococcus aureus* biofilms: properties, regulation, and roles in human disease. Virulence 2:445–459. doi:10.4161/viru.2.5.17724. 21921685 PMC3322633

[5] Yahav D, Rozen-Zvi B, Gafter-Gvili A, Leibovici L, Gafter U, Paul M. 2008. Antimicrobial lock solutions for the prevention of infections associated with intravascular catheters in patients undergoing hemodialysis: systematic review and meta-analysis of randomized, controlled trials. Clin Infect Dis 47:83–93. doi:10.1086/588667. 18498236

[6] Sharan M, Vijay D, Dhaka P, Bedi JS, Gill JPS. 2022. Biofilms as a microbial hazard in the food industry: A scoping review. J Appl Microbiol 133:2210–2234. doi:10.1111/jam.15766. 35945912

[7] Denissen J, Reyneke B, Waso-Reyneke M, Havenga B, Barnard T, Khan S, Khan W. 2022. Prevalence of ESKAPE pathogens in the environment: antibiotic resistance status, community-acquired infection and risk to human health. Int J Hyg Environ Health 244:114006. doi:10.1016/j.ijheh.2022.114006. 35841823

[8] Warrier A, Satyamoorthy K, Murali TS. 2021. Quorum-sensing regulation of virulence factors in bacterial biofilm. Future Microbiol 16:1003–1021. doi:10.2217/fmb-2020-0301. 34414776

[9] Galar A, Weil AA, Dudzinski DM, Munoz P, Siedner MJ. 2019. Methicillin-resistant *Staphylococcus aureus* prosthetic valve endocarditis: pathophysiology, epidemiology, clinical presentation. Diagnosis, and Management Clin Microbiol Rev 32:e00041-18. doi:10.1128/CMR.00041-18. 30760474 PMC6431130

[10] Aslam B, Wang W, Arshad MI, Khurshid M, Muzammil S, Rasool MH, Nisar MA, Alvi RF, Aslam MA, Qamar MU, Salamat MKF, Baloch Z. 2018. Antibiotic resistance: a rundown of a global crisis. Infect Drug Resist 11:1645–1658. doi:10.2147/IDR.S173867. 30349322 PMC6188119

[11] Roy R, Tiwari M, Donelli G, Tiwari V. 2018. Strategies for combating bacterial biofilms: a focus on anti-biofilm agents and their mechanisms of action. Virulence 9:522–554. doi:10.1080/21505594.2017.1313372. 28362216 PMC5955472

[12] Singh L, Cariappa MP, Kaur M. 2016. *Klebsiella oxytoca*: an emerging pathogen? Med J Armed Forces India 72:S59–S61. doi:10.1016/j.mjafi.2016.05.002. 28050072 PMC5192185

[13] Hoenigl M, Valentin T, Zarfel G, Wuerstl B, Leitner E, Salzer HJ, Posch J, Krause R, Grisold AJ. 2012. Nosocomial outbreak of *Klebsiella pneumoniae* carbapenemase-producing *Klebsiella oxytoca* in Austria. Antimicrob Agents Chemother 56:2158–2161. doi:10.1128/AAC.05440-11. 22290949 PMC3318378

[14] Levy Hara G, Gould I, Endimiani A, Pardo PR, Daikos G, Hsueh PR, Mehtar S, Petrikkos G, Casellas JM, Daciuk L, Paciel D, Novelli A, Saginur R, Pryluka D, Medina J, Savio E. 2013. Detection, treatment, and prevention of carbapenemase-producing Enterobacteriaceae: recommendations from an International Working Group. J Chemother 25:129–140. doi:10.1179/1973947812Y.0000000062. 23783137

[15] Leitner E, Zarfel G, Luxner J, Herzog K, Pekard-Amenitsch S, Hoenigl M, Valentin T, Feierl G, Grisold AJ, Hogenauer C, Sill H, Krause R, Zollner-Schwetz I. 2015. Contaminated handwashing sinks as the source of a clonal outbreak of KPC-2-producing *Klebsiella oxytoca* on a hematology ward. Antimicrob Agents Chemother 59:714–716. doi:10.1128/AAC.04306-14. 25348541 PMC4291428

[16] Roszczenko P, Szewczyk OK, Czarnomysy R, Bielawski K, Bielawska A. 2022. Biosynthesized gold, silver, palladium, platinum, copper, and other transition metal nanoparticles. Pharmaceutics 14:2286. doi:10.3390/pharmaceutics14112286. 36365105 PMC9692384

[17] Shkodenko L, Kassirov I, Koshel E. 2020. Metal oxide nanoparticles against bacterial biofilms: perspectives and limitations. Microorganisms 8:1545. doi:10.3390/microorganisms8101545. 33036373 PMC7601517

[18] Król A, Pomastowski P, Rafińska K, Railean-Plugaru V, Buszewski B. 2017. Zinc oxide nanoparticles: synthesis, antiseptic activity and toxicity mechanism. Adv Colloid Interface Sci 249:37–52. doi:10.1016/j.cis.2017.07.033. 28923702

[19] Siddiqi KS, ur Rahman A, Tajuddin A, Husen A. 2018. Properties of zinc oxide nanoparticles and their activity against microbes. Nanoscale Res Lett 13:141. doi:10.1186/s11671-018-2532-3. 29740719 PMC5940970

[20] La Porta FA, Gracia L, Andres J, Sambrano JR, Varela JA, Longo E. 2014. A DFT study of structural and electronic properties of ZnS polymorphs and its pressure-induced phase transitions. J Am Ceram Soc 97:4011–4018. doi:10.1111/jace.13191.

[21] Savu R, Parra R, Joanni E, Jancar B, Eliziario SA, de Camargo R, Bueno PR, Varela JA, Longo E, Zaghete MA. 2009. The effect of cooling rate during hydrothermal synthesis of ZnO nanorods. J Crystal Growth 311:4102–4108. doi:10.1016/j.jcrysgro.2009.06.039.

[22] Kumar R, Umar A, Kumar G, Nalwa HS. 2017. Antimicrobial properties of ZnO nanomaterials: a review. Ceramics International 43:3940–3961. doi:10.1016/j.ceramint.2016.12.062.

[23] Nino-Martinez N, Salas Orozco MF, Martinez-Castanon GA, Torres Mendez F, Ruiz F. 2019. Molecular mechanisms of bacterial resistance to metal and metal oxide nanoparticles. Int J Mol Sci 20. doi:10.3390/ijms20112808. PMC6600416 31181755

[24] Hachicho N, Hoffmann P, Ahlert K, Heipieper HJ. 2014. Effect of silver nanoparticles and silver ions on growth and adaptive response mechanisms of *Pseudomonas putida* mt-2. FEMS Microbiol Lett 355:71–77. doi:10.1111/1574-6968.12460. 24801753

[25] Panáček A, Kvítek L, Smékalová M, Večeřová R, Kolář M, Röderová M, Dyčka F, Šebela M, Prucek R, Tomanec O, Zbořil R. 2018. Bacterial resistance to silver nanoparticles and how to overcome it. Nature Nanotech 13:65–71. doi:10.1038/s41565-017-0013-y. 29203912

[26] Ahmed FY, Aly UF, Abd El-Baky RM, Waly N. 2021. Effect of titanium dioxide nanoparticles on the expression of efflux pump and quorum-sensing genes in MDR *Pseudomonas aeruginosa* isolates. Antibiotics (Basel) 10:625. doi:10.3390/antibiotics10060625. 34073802 PMC8225175

[27] Finley PJ, Norton R, Austin C, Mitchell A, Zank S, Durham P. 2015. Unprecedented silver resistance in clinically isolated enterobacteriaceae: major implications for burn and wound management. Antimicrob Agents Chemother 59:4734–4741. doi:10.1128/AAC.00026-15. 26014954 PMC4505248

[28] Li XZ, Nikaido H, Williams KE. 1997. Silver-resistant mutants of *Escherichia coli* display active efflux of Ag+ and are deficient in porins. J Bacteriol 179:6127–6132. doi:10.1128/jb.179.19.6127-6132.1997. 9324262 PMC179518

[29] Jakovljevic V, Grujic S, Simic Z, Ostojic A, Radojevic I. 2022. Finding the best combination of autochthonous microorganisms with the most effective biosorption ability for heavy metals removal from wastewater. Front Microbiol 13:1017372. doi:10.3389/fmicb.2022.1017372. 36267171 PMC9577556

[30] Prema D, Binu NM, Prakash J, Venkatasubbu GD. 2021. Photo induced mechanistic activity of GO/Zn(Cu)O nanocomposite against infectious pathogens: potential application in wound healing. Photodiagnosis Photodyn Ther 34:102291. doi:10.1016/j.pdpdt.2021.102291. 33862280

[31] Rosenberg M, Visnapuu M, Vija H, Kisand V, Kasemets K, Kahru A, Ivask A. 2020. Selective antibiofilm properties and biocompatibility of nano-ZnO and nano-ZnO/Ag coated surfaces. Sci Rep 10:13478. doi:10.1038/s41598-020-70169-w. 32778787 PMC7417576

[32] Ghasemian A, Mobarez AM, Peerayeh SN, Bezmin Abadi AT. 2019. The association of surface adhesin genes and the biofilm formation among Klebsiella oxytoca clinical isolates. New Microbes New Infect 27:36–39. doi:10.1016/j.nmni.2018.07.001. 30581573 PMC6290254

[33] Garcia-Lara B, Saucedo-Mora MA, Roldan-Sanchez JA, Perez-Eretza B, Ramasamy M, Lee J, Coria-Jimenez R, Tapia M, Varela-Guerrero V, Garcia-Contreras R. 2015. Inhibition of quorum-sensing-dependent virulence factors and biofilm formation of clinical and environmental Pseudomonas aeruginosa strains by ZnO nanoparticles. Lett Appl Microbiol 61:299–305. doi:10.1111/lam.12456. 26084709

[34] Kudaer NB, Risan MH, Yousif E, Kadhom M, Raheem R, Salman I. 2022. Effect of zinc oxide nanoparticles on capsular gene expression in *Klebsiella pneumoniae* isolated from clinical samples. Biomimetics 7:180. doi:10.3390/biomimetics7040180. 36412708 PMC9680528

[35] Saleh MM, Sadeq RA, Latif HKA, Abbas HA, Askoura M. 2019. Zinc oxide nanoparticles inhibits quorum sensing and virulence in Pseudomonas aeruginosa. Afr H Sci 19:2043–2055. doi:10.4314/ahs.v19i2.28. PMC6794539 31656488

[36] Abd El-Hamid MI, Y El-Naenaeey ES, M Kandeel T, Hegazy WAH, Mosbah RA, Nassar MS, Bakhrebah MA, Abdulaal WH, Alhakamy NA, Bendary MM. 2020. Promising antibiofilm agents: recent breakthrough against biofilm producing methicillin-resistant *Staphylococcus aureus*. Antibiotics (Basel) 9. doi:10.3390/antibiotics9100667. PMC7600973 33022915

[37] CLSI. 2015. M07-A10: Methods for dilution antimicrobial susceptibility tests for bacteria that grow aerobically, p 15–50; Approved Standard - Tenth edition. Wayne, PA.

[38] Orsinger-Jacobsen SJ, Patel SS, Vellozzi EM, Gialanella P, Nimrichter L, Miranda K, Martinez LR. 2013. Use of a stainless steel washer platform to study *Acinetobacter baumannii* adhesion and biofilm formation on abiotic surfaces. Microbiology (Reading) 159:2594–2604. doi:10.1099/mic.0.068825-0. 24025603 PMC3853682

[39] La Porta FA, Andres J, Li MS, Sambrano JR, Varela JA, Longo E. 2014. Zinc blende versus wurtzite ZnS nanoparticles: control of the phase and optical properties by tetrabutylammonium hydroxide. Phys Chem Chem Phys 16:20127–20137. doi:10.1039/c4cp02611j. 25133930

